# Quantification of Cytomegalovirus Glycoprotein Bn DNA in Hematopoietic Stem Cell Transplant Recipients by Real-time PCR

**DOI:** 10.1371/journal.pone.0051224

**Published:** 2012-12-10

**Authors:** Xuan Zhang, Ya Ping Huang, Hai Nv Gao, Mei Fang Yang, Hong Zhao, Jian Hua Hu, Xiao Ming Chen, Wei Hang Ma, Jun Fan

**Affiliations:** State Key Laboratory for Diagnosis and Treatment of Infectious Diseases, First Affiliated Hospital, Zhejiang University School of Medicine, Hangzhou, China; University Hospital San Giovanni Battista di Torino, Italy

## Abstract

Based on sequence variation in the N-terminus of the UL55 gene, which encodes glycoprotein B (gB), human cytomegalovirus (CMV) can be classified into four gBn genotypes. We assessed the distribution of CMV gBn genotypes and the correlation between CMV gBn DNA (detected by real-time PCR) and CMV-positive pp65 cells (identified by immunohistochemical staining) in a cohort of hematopoietic stem cell transplant patients. The distribution of gB genotypes was as follows: gBn1, 60% of patients; gBn2, 13.3%; mixed gBn1 and gBn3 infection, 26.7%; and gBn4 and other mixed infections, 0%. CMV gBn1 was the most common genotype. The detected level of CMV gB DNA correlated well with the number of CMV-positive pp65 cells detected by immunostaining (r = 0.585).

## Introduction

Cytomegalovirus (CMV) continues to be a major cause of morbidity and mortality in hematopoietic stem cell transplant (HSCT) patients, despite recent advances in the development of antiviral drugs and diagnostic techniques [1]. CMV glycoprotein B (gB) is the major envelope glycoprotein of CMV and is encoded by the UL55 gene. CMV gB has been implicated in host cell entry, cell-to-cell virus transmission, and fusion of infected cells, in addition to its role as an important target of both the humoral and cellular immune responses [2], [3]. CMV gB is expressed as a precursor molecule that is glycosylated and then cleaved between amino acid residues 460 and 461 to form a disulfide-linked complex of gp55 and gp116 [4]. Peptide variations in gp116 are strongly clustered in specific regions of the protein. Analyses of fragments corresponding to the N-terminus (gBn) of gp116 or the cleavage site (gBcls) and C-terminus (gBc) of gp55 have identified four gBn and gBcls genotypes and two gBc genotypes [4], [5].

We determined the distribution of CMV gBn genotypes in a Chinese population of HSCT recipients and examined the correlations among gBn genotype, pp65 antigen and CMV gBn DNA.

## Methods

### Patients and Samples

This study included 27 recipients who were followed for more than 6 months after hematopoietic stem cell transplantation at the First Affiliated Hospital of Zhejiang University School of Medicine between April, 2009 and June, 2010. Ethylenediaminetetraacetic acid (EDTA)-treated blood samples were collected at 3 and 6 months after transplantation for the detection of CMV pp65 antigen, CMV gBn genotype and gBn DNA as described below. CMV pp65 antigen results were used to make clinical decisions.

### Ethics Statement

Informed consent was obtained from all patients, and the local ethics committee, the Medical ethics committee of the First Affiliated Hospital, College of Medicine, Zhejiang University, approved the study, which conformed to the ethical guidelines of the 1975 Helsinki Declaration.

### Immunohistochemical Staining [6], [7]

A standard two-step immunohistochemical method was used to detect CMV antigen expression in peripheral blood leukocytes. In brief, leukocytes were separated from EDTA- treated blood and were spread on slides. Anti-CMV-PP65-Ag monoclonal antibody (DAKO, Denmark) and an EnVision+™ system peroxidase (DAB) kit (DAKO) were used to stain CMV antigen on the slides. The stained samples were visualized under an optical microscope, using an imaging recording system (BH-2, Olympus, Japan). Cells staining yellow or brown were positive, and blue cells were negative. The results are reported as the number of positive cells per 50,000 leukocytes.

### gB PCR and Genotyping

CMV gBn genotyping by real-time quantitative PCR was successfully established in our previous study [8]. The blood samples, from patients infected with EBV, HBV, HCV and HHV-6, were detected by real-time quantitative PCR. The results were negative.

### Statistical Analysis

The statistical analysis was performed using SPSS ver. 11.5. Categorical variables were analyzed using a *t*-test. One-way analysis of variance was used to compare the CMV gBn DNA of HSCT patients with the different CMV gBn genotypes. Spearman’s correlation coefficient was calculated to compare the amount of CMV gBn DNA and the number of CMV-positive pp65 cells. Differences were considered significant at *p*<0.05.

## Results

### Patient Demographics

Fifty-four EDTA-treated blood samples from 27 HSCT patients were examined for CMV pp65 antigen, CMV gBn genotype, and gBn DNA. The demographic characteristics of the 27 patients are shown in Table 1.

**Table 1:** The demographic and clinical characteristics of 27 HSCT patients (54 samples).

Characteristic		
Mean age (year) (95%confidence interval)	24.04±7.93 (20.90–27.17)	
Sex (female:male)	16∶11	
underlying diseases		
acute lymphoblastic leukemia	5	
acute myeloid leukemia	12	
chronic myeloid leukemia	8	
Other diseases	2	
Pp65 cells,no of positive samples	15 (27.8%)	
CMV gB DNA,no.of positive samples	48 (88.9%)	
	3month	6month
Positive pp65 cells,no of patients	25 (92.6%)	23 (85.2%)
Single CMV gB genotype,no.of patients	4	7
Mixed CMV gB genotypes,no.of patients	3	1

### CMV pp65 Antigen

**Figure 1:**
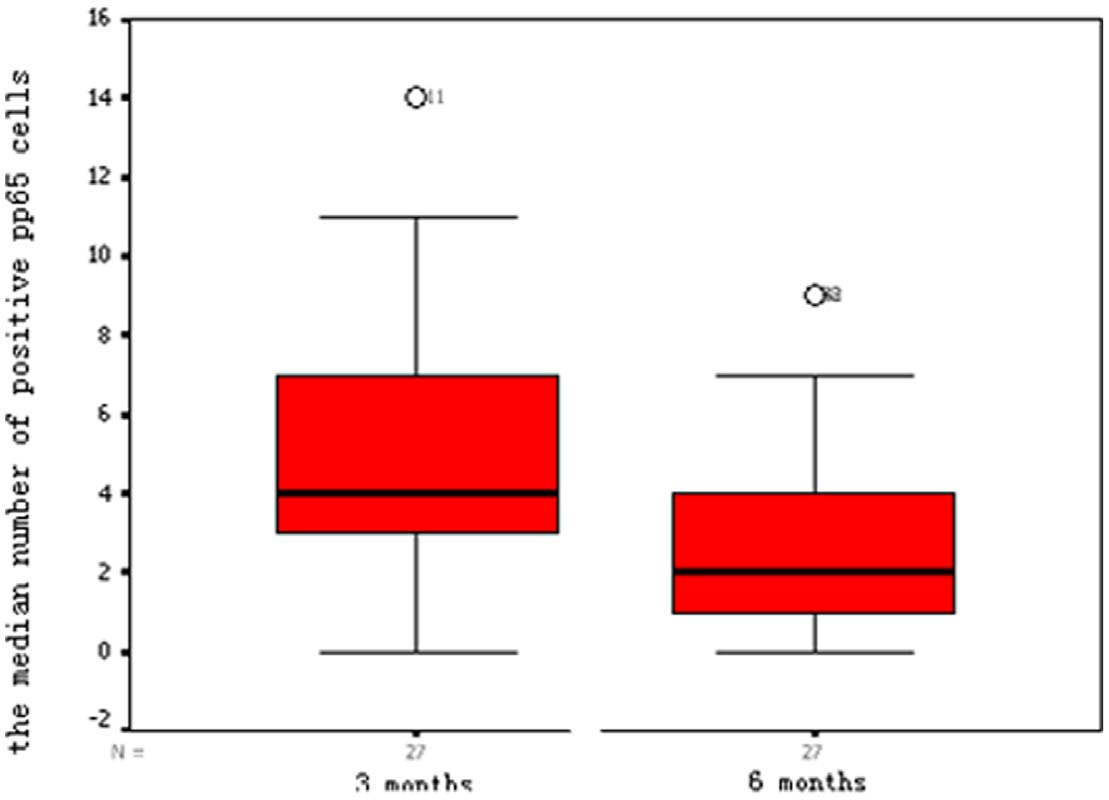
The median number of CMV pp65 positive cells per 50,000 leukocytes at 3 months and 6 months post-transplantation.

Cytomegalovirus pp65 antigen was found in 48 (88.9%) of the 54 blood samples. Three months after transplantation, 25 of 27 samples were positive, with a median of four positive cells (range, 0–14), which was significantly higher than the number present at 6 months post-transplantation (median, 2 cells; range, 0–9; Figure 1).

### CMV gBn Genotypes and DNA

Quantitative detection by real-time PCR revealed the CMV gBn genotype in 15 samples (seven 3-month samples and eight 6-month samples) from 13 patients, representing 27.8% of the 54 plasma samples.

**Figure 2:**
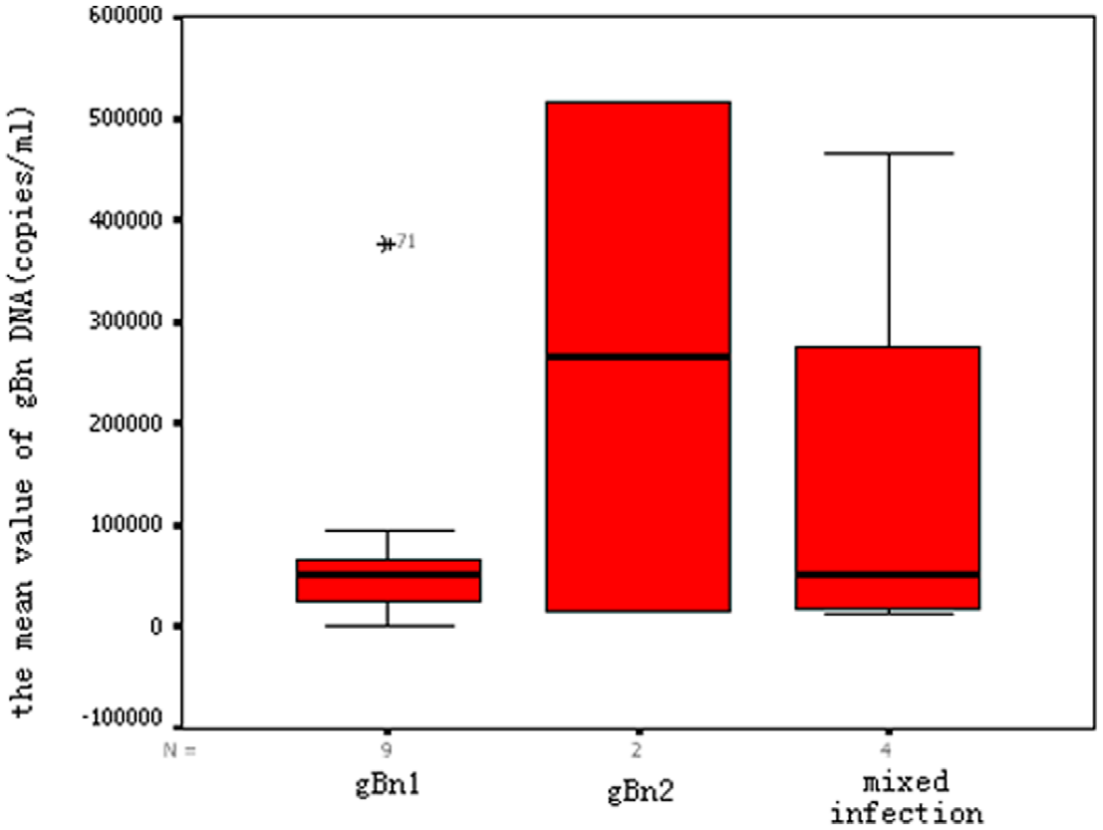
CMV gBn DNA (copies/ml) in HSCT patients within each of the CMV gB genotype groups.

The distribution of CMV gBn genotypes for all 15 samples was as follows: gBn1, nine (60.0%); gBn2, two (13.3%); and mixed gBn3 and gBn1, four (26.7%). The gBn4 genotype was not detected in any sample. Real-time PCR was used to quantitatively determine the mean CMV gBn DNA load for each genotype, with the following results (expressed as copies/mL): gBn1, 80,400 (range, 1,790–377,000); gBn2, 265,000 (range, 15,800–515,000); gB1 and gBn3 mixed infection, 146,000 (range, 12,500–466,000) (log_10_ gB DNA, F = 0.360, *p* = 0.705 for differences between groups; Figure 2). The mean virus load for a single gBn genotype infection was 114,000 copies/mL (range, 1,790–515,000 copies/mL), which was lower, but not significantly lower (log_10_ gB DNA, *p* = 0.700), than that for the mixed infection.

Among the seven 3-month samples, there were four gBn1 genotypes (57.1%) and three mixed gBn1 and gBn3 genotypes (43.9%). The genotypes present in the eight 6-month samples comprised five gBn1 genotypes (62.5%), two gB2 genotypes (25.0%) and one mixed gBn1 and gBn3 genotype (12.5%). In addition, the mean virus load was higher at 6 months than at 3 months post-transplantation (190,000 *vs*. 45,400 copies/mL), although the difference was not significant (log_10_ gB DNA, *p* = 0.360).

### CMV gBn DNA and pp65

**Figure 3:**
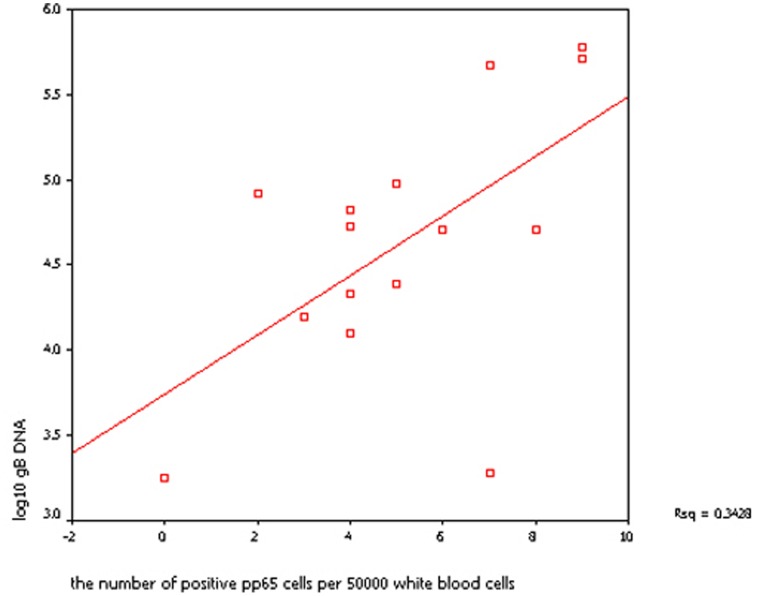
The relation of CMV gBn DNA (log10 DNA) and the number of positive pp65 cells per 50000 white blood cells in HSCT patients (r^2^ = 0.3428).

Of the 54 blood samples, 48 contained pp65-positive cells, and 15 of those samples also contained gBn DNA. Among these 15 samples, the mean number of pp65-positive cells in the 3-month samples was 4 (range, 0–8), which was lower than the number in the samples taken at 6 months post-transplantation (median, 6 cells; range, 3–9) but not significantly so (*p* = 0.169). The mean gBn DNA content was also higher in the 6-month samples than in the 3-month samples (190,000 *vs*. 45,400 copies/mL), although the difference was not significant (log_10_ gB DNA, *p* = 0.360). The level of CMV gB DNA detected by real-time PCR correlated well with the number of pp65-positive cells determined by immunohistochemical staining (log_10_ gB DNA, r = 0.585, r^2^ = 0.3428, *p*<0.05, Spearman’s correlation coefficient; Figure 3).

## Discussion

CMV infection is common and 40–100% of the population have anti-CMV antibodies in their serum [7]. Reactivation of CMV is the third main cause of mortality in transplant patients during the first three postoperative months [7], [9]. In this study, CMV gBn genotypes were detected by real-time quantitative PCR. The gBn genotype was identified in 15 plasma samples from 13 of 27 Chinese HSCT recipients. Among the 15 samples, gBn1 was the most common genotype (60%), gBn2 was relatively uncommon (13.3%), and gBn3 was found only in mixed infections with gBn1 (26.7%). gBn4 and other mixed infections were not found.

Many studies have reported the distribution of CMV gB genotypes in immunocompromised patients. Torok–Storb *et al*. [10] reported that in 281 bone marrow transplantation recipients with CMV infection, the distribution of gB types 1–4 was 48.4, 16.4, 24.6, and 8.2%, respectively, with only 2.5% of all isolates containing more than one gB type. The dominance of the gB1 genotype in congenital human CMV infections was reported in a population from southern Hungary [11]. The distribution of the gB genotypes in our study was similar those in previous reports. Note that we grouped gB using the N-terminus of gp116, whereas CMV gB types in previous studies were always grouped by cleavage site. Many factors, including differences in the underlying disease, race, and detection technology, can explain the variation in the geographical distribution of CMV genotypes.

We found that the CMV gBn DNA content did not differ significantly among the gB genotypes. Furthermore, the level of CMV gBn DNA detected by real-time PCR correlated well with the number of CMV pp65-positive cells detected by immunostaining. Both the number of pp65-positive cells and the mean gBn DNA content were higher in the 6-month samples than in the 3-month samples. We consider that this was due to antiviral drug use.

Currently, many virology laboratories use the pp65 antigenemia assay as the gold standard for evaluating or validating in-house molecular methodologies [12]. The assay can be used to predict CMV disease, as a higher level of antigenemia has a higher predictive value for disease in all patient groups [13]. The CMV antigenemia assay has been used for pre-emptive therapy with considerable success [14]. We found a good correlation between the level of CMV gB DNA and the number of CMV pp65-positive cells. Therefore, we postulate that the CMV gBn genotype and DNA load may help in both predicting CMV infection and disease and in guiding pre-emptive therapy.

Among the 54 samples, 15 (27.8%)were found to be positive by real-time quantitative PCR, whereas 48(88.9%) were found to be positive by pp65 antigenemia assay. In HCMV reproduction, the synthesis of pp65 precedes that of gB. In addition, all recipients in this study received antivirus therapy after transplantation, which affected the gBn DNA detection rate.

In summary, CMV gBn genotypes were detected in 13 of 27 Chinese HSCT recipients. We also showed that the level of CMV gB DNA detected by real-time PCR correlated well with the number of CMV pp65-positive cells identified by immunostaining. Currently, CMV glycoprotein DNA is researched only rarely, and the application of antiviral drugs affects detection of DNA. Therefore, we infer that detection of CMV glycoprotein B DNA may facilitate assessment of treatment of CMV infection. In future, studies of the relationship between specific CMV gB types and clinical outcome in immunocompromised patients, and of pre-emptive therapy guided by the level of CMV gBn DNA, should help in the diagnosis and treatment of CMV-associated disease.
